# Safety and feasibility of the RhinoChill immediate transnasal evaporative cooling device during out-of-hospital cardiopulmonary resuscitation: A single-center, observational study

**DOI:** 10.1097/MD.0000000000004692

**Published:** 2016-08-26

**Authors:** Marie-Sophie Grave, Fritz Sterz, Alexander Nürnberger, Stergios Fykatas, Mathias Gatterbauer, Albert Friedrich Stättermayer, Andreas Zajicek, Reinhard Malzer, Dieter Sebald, Raphael van Tulder

**Affiliations:** a Department of Emergency Medicine, Medical University of Vienna; b Wiener Berufsrettung, Municipal Ambulance Service, Vienna; c University Hospital St. Pölten, Karl-Landsteiner Medical University, Lower Austria; d Department of Internal Medicine III, Divison of Gastroenterology and Hepatology, Medical University of Vienna, Vienna, Austria.

**Keywords:** airway management, cardiopulmonary resuscitation, feasibility studies, hypothermia, induced, nasopharynx, safety

## Abstract

We investigated feasibility and safety of the RhinoChill (RC) transnasal cooling system initiated before achieving a protected airway during cardiopulmonary resuscitation (CPR) in a prehospital setting.

In out-of-hospital cardiac arrest (OHCA), transnasal evaporative cooling was initiated during CPR, before a protected airway was established and continued until either the patient was declared dead, standard institutional systemic cooling methods were implemented or cooling supply was empty. Patients were monitored throughout the hypothermia period until either death or hospital discharge. Clinical assessments and relevant adverse events (AEs) were documented over this period of time.

In total 21 patients were included. Four were excluded due to user errors or meeting exclusion criteria. Finally, 17 patients (f = 6; mean age 65.5 years, CI95%: 57.7–73.4) were analyzed. Device-related AEs, like epistaxis or nose whitening, occurred in 2 patients. They were mild and had no consequence on the patient's outcome. According to the field reports of the emergency medical services (EMS) personnel, no severe technical problems occurred by using the RC device that led to a delay or the impairment of quality of the CPR.

Early application of the RC device, during OHCA is feasible, safe, easy to handle, and does not delay or hinder CPR, or establishment of a secure intubation. For efficacy and further safety data additional studies will be needed.

## 1 Introduction

The use of therapeutic hypothermia after cardiac arrest (CA) has been shown to improve neurologic outcome.^[1,2]^ Because the cascade of metabolic damage occurs within the 1st minutes during CA^[3,4]^ it has been postulated that hypothermia should be induced as soon as possible,^[5]^ possibly within the 1st minutes during on-going cardiopulmonary resuscitation (CPR). Moreover, animal studies suggest that intraarrest cooling might be beneficial^[6–8]^ to reduce the nonperfusion and reperfusion damage summarized under the term “postcardiac arrest syndrome.”^[9]^ The on-going discussion moves from “whom do we cool” to “when do we start to cool.” Although a large amount of scientific work concerns therapeutic hypothermia, the exact window to initiate cooling is still unknown but hypothermia induction glides more and more into the field of emergency medical services (EMS).

At the moment in-hospital cooling is frequently accomplished by invasive systems with intravascular cannulas but also noninvasive by application of external cooling pads, both frequently assisted by rapid infusion of ice-cold saline.^[10]^ Out-of-hospital induction of hypothermia is more complex and still there is no evidence that inducing hypothermia in the prehospital setting is beneficial. Therefore, out-of-hospital hypothermia in our system is mostly induced by the application of cool packs alone. Nevertheless prehospital induction of therapeutic hypothermia by EMS personnel is limited not only because of space restrictions for nowadays available cooling devices but also by our ability to cool rapidly with the available techniques of ice packs and cold intravenous saline. Furthermore, rapid intraarrest cooling seems not to be feasible by application of cool packs as efficacy of heat transfer is limited.^[11]^

The RhinoChill (RC, BeneChill Inc. San Diego, CA) intranasal cooling device has been specifically designed for a prehospital use. Prior conducted studies showed that the use of this device is safe, which means the patients were not adversely affected. One in-hospital trial showed that it could be easily integrated into a clinical setting, and that cooling of the brain is established rapidly while the body is cooled more slowly.^[11]^ In the PRINCE study, a multicenter trial, RC was induced after a protected airway has been established.^[12]^ Although this study was not intended and powered to show differences in outcome a trend toward improved survival with good neurological outcome was noticed.

The current study was set to investigate safety and feasibility of the RC device for intraarrest cooling prior to a protected airway in an out-of-hospital cardiac arrest (OHCA).

## 2 Methods

### 2.1 Study design

This study was designed as a prospective single-center, single-arm observational, and investigator initiated feasibility study, conducted by emergency physicians in OHCA. The study was performed from December 2011 to December 2013.

We aimed to demonstrate that the application of RC “as soon as possible” in OHCA is feasible and safe, and quality of CPR does not suffer from handling this new device in any way. Ethical consideration for enrolling patients without their consent were in accordance with the World Medical Association Helsinki Declaration of 1964, as revised at the 52nd General Assembly in Edinburgh in 2000, and our responsible committee on human experimentation (EK Nr: 745/2011).

### 2.2 Study setting

The study was performed at the Vienna Municipal Ambulance Service. In a 2-tiered system with both physician and paramedic response 45 ambulances and 12 emergency physician vehicles cover 1.7 million inhabitants. Approximately 150,000 emergency runs per year are conducted by this system.

### 2.3 The cooling device

The RC transnasal cooling system is a portable device, which runs on batteries. Cooling is established by spraying an evaporative coolant (perfluorohexane [PFH]) into the nasal cavity via intranasal cannulas. The coolant is a colorless, odorless, and radiolucent liquid. The RC device takes advantage of the nose as a natural orifice into the head to overcome the obstacle of cooling through the scull. By spraying the coolant into the large diffuse surface area of the upper nasal pathway, heat is absorbed from the tissue and thereby cooling the tissue and the innate vasculature that supplies blood to the brain. Cooling via the nasopharynx therefore offers the ability to cool across the thin cribriform plate via both direct conductive mechanisms that require blood circulation as well as hematogenous mechanisms that do.^[13]^ Local temperatures within the nasal cavity are expected to cool down to approximately 2 °C.

### 2.4 Patient population

Patients suffering from OHCA, older than 18 years under EMS initiated CPR were included into this study. Traumatic etiology of CA, obvious pulmonary disease, intranasal cannulas not fully advance-able, pregnancy, known coagulopathy, and an already protected airway served as exclusion criteria.

### 2.5 Performance

Ten RC devices had been available on 5 emergency bases of the Municipal Ambulance Service, Vienna. Twelve dedicated emergency physicians were trained in the application of RC. The emergency physicians were the only ones to enrol patients (no usage by paramedics due to legal consideration). After inclusion the emergency physician placed the RC intranasal cannulas and began to cool during on-going standard resuscitation procedures as soon as possible. “As soon as possible” means placing the cannulas before intubation (endotracheal or laryngeal tube), but already during chest compressions and bag valve mask ventilation. Standard life-support procedures should not be delayed in any way and were not due to the field reports. The time to place the cannulas took a short period of time and ventilation pauses due to chest compressions could be therefore used. Cooling with RC was performed until either the coolant container was empty or the patient was declared dead on-scene.

### 2.6 Data collection

After using an RC device in a rescue effort physicians were asked to complete a protocol asking on the patients’ demographic (e.g., initials, sex, and date of birth), inclusion and exclusion criteria, the situation on-site (e.g., the place, witnessed/nonwitnessed CA, and 1st [electrocardiogram]-rhythm), and details of cooling (e.g., time cooling was started, duration, problems with running the RC device, and adverse events [AEs]).

All surviving patients underwent standard postresuscitation care protocols upon hospital arrival per the institution's standard operating procedures. All clinically significant general AEs (risks considered being associated with the use of the device as well as those related to the hypothermia itself) over the 1st 24 hours had to be reported and all new onset serious adverse events occurring after hospital admission had to be recorded throughout the 1st 7 days of hospitalization. The nature and severity of the AE, the relationship to the RC device, management, and outcome were recorded. Based on the analysis of the continuous electrocardiogram and impedance data, we also evaluated the quality of CPR performed by emergency medical technicians.

All data were prospectively collected and double entered by trained data assessors into an Excel Database (MS Excel for Mac, Microsoft, Redmond, WA).

### 2.7 Statistical analysis

Descriptive statistics are calculated for all performance, safety, demographic, and baseline variables. Means, confidence intervals (95%), and ranges are used to describe continuous measurements. Counts and percentages were used to describe categorical parameters. All statistical analyses were carried out using a commercially available software system (SPSS for Windows, version 21, IBM Corporation, NY).

## 3 Results

**Figure 1:**
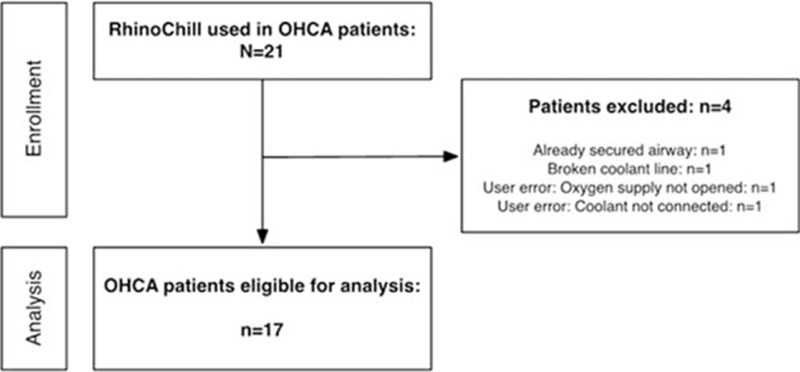
Participants flowchart.

In the study period of 24 months, emergency physicians screened 21 patients without signs of circulation. A flowchart is given in Fig. 1. Demographic data are summarized in Table 1. From these 21 patients, 4 patients had to be excluded from final analysis, the reasons are shown in Fig. 1 and Table 1. Finally, 17 patients were eligible for analysis. A flowchart of all patient's out-of-hospital characteristics is given in Table 2.

**Table 1:**
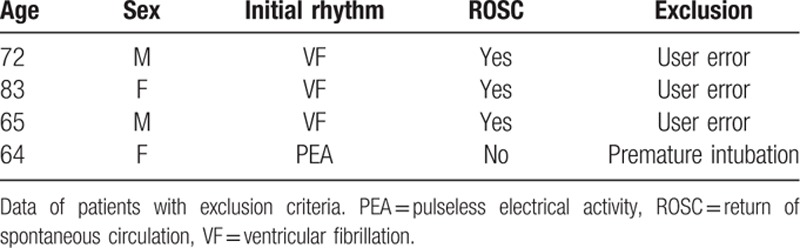
Excluded out-of-hospital cardiac arrest patients.

**Table 2:**
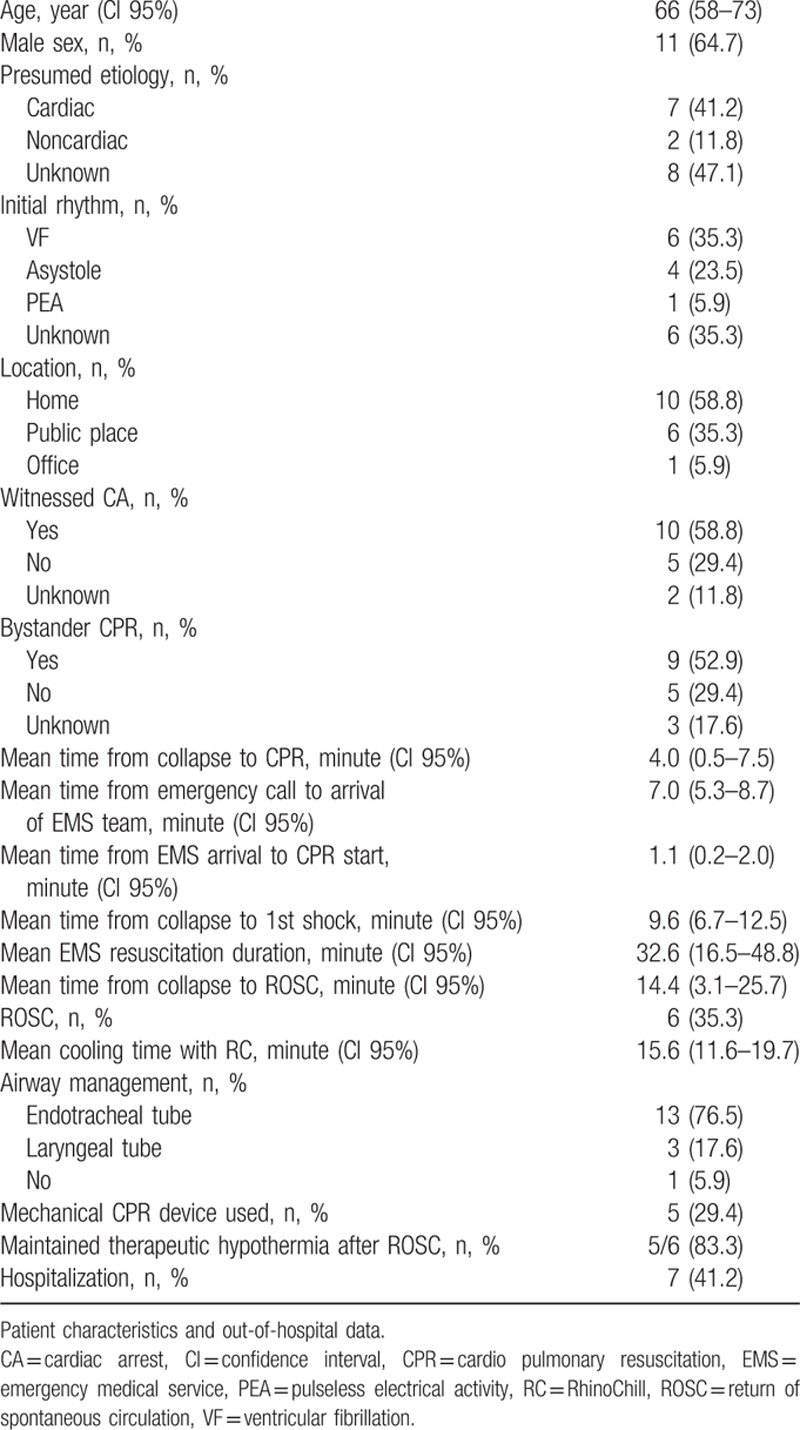
Characteristics of out-of-hospital cardiac arrest patients.

### 3.1 Adverse events

AEs, in connection to the use of the RC device, which were manufacturer, described a priori included epistaxis, cold-related tissue damage, reversible discoloration of the nasal tip, emphysema (periorbital, pulmonary), hypertension, hypoxia, and over-pressure injury in the nasal cavity/nasopharynx. In our study, AEs occurred in 2 patients (11.8%). They were mild and reversible. In 1 patient, the cooling with the RC device caused nose whitening, which also stopped spontaneously after finishing the treatment with the cooling device. In another patient mild epistaxis occurred, which also suspended spontaneously. This and the unfortunately low number of patients did not allow long-term follow-ups with regards to additional AEs provoked by nasopharyngeal cooling.

### 3.2 Quality of CPR

Quality measures of CPR were monitored and are shown in Table 3. Performance values were within guidelines recommendations.

**Table 3:**
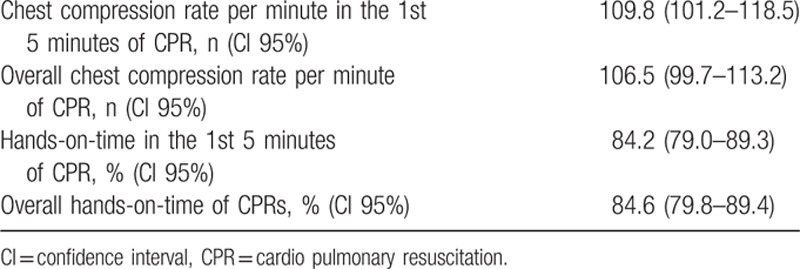
Quality measures of performed CPR.

### 3.3 Handling and technical problems

**Figure 2:**
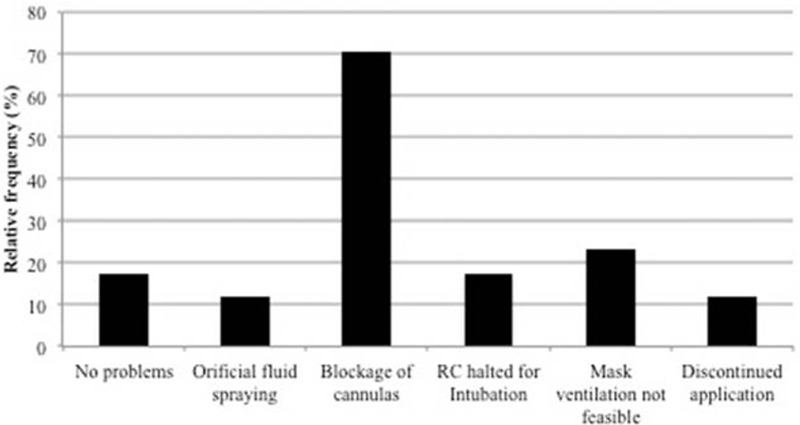
Relative frequency of technical problems in using the RhinoChill (RC) device.

In general, emergency physicians rated handling of the RC device as simple. Implementation of the RC device in the field was easy, and potential technical problems could be handled. According to the filed reports of the emergency physicians, the technical problems occurred were not rated severe and did not impact quality of the CPR. The most commonly observed interruption in the application was short blockage (for a few seconds) of the device (n = 12, 70.6%). This is a safety precaution to prevent damage of the nasal cavity from the increasing pressure in case of an elevated resistance. On one hand, the blockages tended to happen due to increased pressure during ventilation of the patient with a bag-valve-mask and on the other hand, because the cannulas were in contact with the nasal mucosa, which also led to an increase of resistance. In the case of too close contact, a repositioning of the cannulas helped to solve the problem. In 2 patients (11.8%) the RC usage had to be discontinued due to nonrecoverable blockage. In 4 patients (23.5%) the bag-valve mask-ventilation was disabled because cannulas hampered sealing the mask on the face. In 3 patients (17.6%) the RC device application had to be interrupted for intubation because fluid, caused by the PFH instillation, obscured the view. After intubation the RC application was continued without any problem. In 2 patients (11.8%) a moderate orificial fluid spraying out of the mouth was observed during chest compressions. In 3 patients (17.6%) the device was used without difficulty. Figure 2 shows a summary of technical problems encountered with the RC device use.

## 4 Discussion

This feasibility study is the 1st to show that in OHCA, intraarrest, transnasal, evaporative cooling prior to a secured airway is feasible and safe. The implementation of the RC device simultaneously with the start of CPR did not hinder the standard resuscitation procedure and had no negative influence on the quality of standard resuscitation efforts. AEs rarely happened, were mild, reversible, and had no influence on the patient's outcome. No serious AEs were reported. The majority of technical problems that occurred were simple to identify and in general amendable.

This study was authorized under the permission that only emergency physicians were allowed to use the RC device in the field. Nevertheless we instructed and trained all study-participating emergency physicians and paramedics in the implementation of RC. Although the handling requires not much training, we recognized the importance of trainings at regular intervals to keep proper handling in mind. We assume that user errors are avoidable by repeated training. The most common technical problem was blockage of the cannulas which was successfully resolved in most patients. Only in 2 patients, the blockage cause could not be identified and therefore could not be solved, which led to discontinuation of RC device use.

This study was not powered to detect any differences in outcome measures such as survival or neurological outcome. In addition, this study was not designed to investigate temperature changes and feasibility of inducing hypothermia in the prehospital setting.

The 1st trial of transnasal evaporative cooling in the out-of-hospital setting was the PRINCE-study.^[12]^ In this randomized study, Castrén et al^[12]^ determined the safety, feasibility, and cooling efficacy of prehospital transnasal cooling via RC in humans and explored its effects on neurologically intact survival to hospital discharge. They included 93 patients, with a witnessed CA and an EMS treatment interval within 20 minutes after collapse, into the treatment group and 101 patients to the standard care group. Patients were randomized after a secured airway was established. Compared to our study, we included all types of OHCA patients who met the inclusion criteria, no matter whether the CA was witnessed or not. Another difference was the time point of establishing the RC device. Although the PRINCE study population needed a secured airway before RC usage, our focus lay on the establishment of the device before securing the airway, which meant “as soon as possible.” Furthermore, patients in the PRINCE study, who achieved return of spontaneous circulation (ROSC), were treated with RC in the ambulance unless consciousness was regained or continued until systemic cooling was started in hospital. In our study, RC was used until either resources (PFH, oxygen) were empty (approx. 20 minutes) or the patient was declared dead. This difference in treatment led to the result that the PRINCE-study was able to make clear statements about the cooling efficacy of RC. In our study, it was not our aim to do so. Concerning AEs both studies observed the same ones, reversible nose whitening and mild epistaxis. We also focused on whether the usage of RC led to any delay in standard resuscitation treatment or a decrease of CPR quality, which was not tested in the PRINCE-study. Both studies were also not powered to demonstrate whether an increase of ROSC rate is achievable with the use of early transnasal evaporative cooling.

Wang et al^[14]^ tested the effect of selective brain cooling in a randomized trial in a porcine model of prolonged CA. The results showed that 7 of 8 animals in the hypothermic group (87.5%) and 2 of 8 animals in the control group (25%) (*P* = 0.04) were successfully resuscitated. The mechanism, by which selective brain cooling improves the resuscitative effort and ROSC, is still unclear. Up to now there are no existing trials showing whether it would be possible to achieve higher ROSC rates in humans. Another trial, concerned with the use of RC in the out-of-hospital setting, has been published by a UK group.^[15]^ The aim was also to describe the feasibility of employing it during prehospital resuscitation for OHCA. They also came to the result that RC was easy to set up and to use during resuscitation, and that it did not interfere with standard resuscitation practice.

Compared to other cooling methods, there is no system that can perfectly be applied in the field. Infusions of ice-cold fluids have the advantage of being cheap and ubiquitous available. The cooling efficacy is good, but is not adequate to maintain hypothermia solely.^[10]^ Another disadvantage is the requirement of a built-in fridge in the ambulance car to cool the fluid permanently. And there is still no evidence for the benefit of therapeutic hypothermia induced in the field with cold fluids.^[16]^ Another method to induce therapeutic hypothermia is via surface cooling by using cooling pads.^[17]^ The device operates independently of power supply, and thus it is suitable for out-of-hospital use. This method has also proven to be feasible, safe, and easy to handle. A negative aspect, as seen with RC, is the storage problem with a quite huge cooling box for pads. All methods have their strengths and weaknesses for the usage in the field.

### 4.1 Limitations

The present study has a number of limitations. First of all, the small number of cases, included in this trial, was a major limitation to make statistically significant conclusions. We planned to recruit 20 patients in a period of 1 year. Although 20 patients are not that much, it was a good number to see if the implementation is either possible or not, and if the Ambulance Service of Vienna is willing to work with it. Unfortunately, 21 patients were included at first, but the number of patients gained for further investigations just amounted to 17 patients and took us much longer than 1 year. The acquisition of this small amount of patient was a result of the underlying conditions. One of the participating physicians had to be on duty on an emergency base where RC was available, and the reason of the emergency call had to be a CA. Otherwise RC was not taken to the patient. But in the end, it was sufficient for an initial assessment. Second, since it was not a randomized trial, we could not find out, whether there could be a higher ROSC rate in patients treated with RC compared to a control group. Third, we could not gain any data on the cooling efficacy of RC, because RC has been stopped after being empty and was not used for further cooling. In fact, this study was not powered to determine the cooling efficacy of RC. Another limitation is the accuracy of data collection. The timing of resuscitation efforts was usually documented after the rescue effort, and therefore it is assumable that the minutes from the point of CA to the implementation of the treatment were not always accurately appreciable. After showing that the implementation of transnasal evaporative cooling via RC device in the out-of-hospital setting of Vienna is feasible, a future aim should be to run a randomized study, where the focus should lie on the question whether it would be possible to achieve higher ROSC rates with the use of transnasal evaporative cooling compared to a control group.

## 5 Conclusion

Early application of transnasal evaporative cooling by using the RC device during OHCA is feasible, safe, easy to handle, and does not delay or hinder CPR, or establishment of a secure airway. For efficacy and further safety and outcome data additional studies will be needed.
